# Nutritional Status of People with a Coexisting Chronic Wound and Extended Assessment Using Bioelectrical Impedance

**DOI:** 10.3390/nu15132869

**Published:** 2023-06-25

**Authors:** Mateusz Skórka, Paweł Więch, Joanna Przybek-Mita, Anna Malisiewicz, Kamila Pytlak, Dariusz Bazaliński

**Affiliations:** 1 St. Hedvig Clinical Provincial Hospital, 35-301 Brzozów, Poland; skorka.mateusz@op.pl (M.S.); annamalisiewicz1@gmail.com (A.M.); 2 Institute of Health Sciences, College of Medical Sciences, University of Rzeszów, 35-959 Brzozów, Poland; joannapm@vp.pl (J.P.-M.); dbazalinski@ur.edu.pl (D.B.); 3 Institute of Health Protection, State University of Applied Sciences in Przemyśl, 37-700 Przemyśl, Poland; 4 Center for Postgraduate Education of Nurses and Midwives, 35-083 Brzozów, Poland; 5 Podkarpackie Specialist Oncology Centre, Specialist Hospital in Brzozów, 36-200 Brzozów, Poland; kamila.pytlak@interia.pl

**Keywords:** bioimpedance, nutrition, malnutrition, nurse, chronic wound

## Abstract

The diagnosis of malnutrition should be one of the pillars of comprehensive patient care, especially in the case of patients with large wounds, prolonged healing, or comorbidities. The condition for a reliable and accurate nutritional diagnosis is to link it with the parameters of nutritional status assessment at the basic level (anthropometric measurements and clinical assessment) and in depth (biochemical tests and bioelectrical impedance). A prospective study included a sample of 60 patients with coexisting chronic wounds (venous ulcers, diabetic foot syndrome, pressure injury) treated at the Wound Treatment Clinic of Fr. B. Markiewicz Podkarpackie Oncology Center (Poland). The method of estimation and diagnostic survey was used; the research tool was a scientific research protocol consisting of four parts. Self-care capacity was assessed based on the Barthel scale, nutritional status using blood biochemical parameters, and electrical bioimpedance. Wounds were classified according to the extent, depth of tissue structures, and potential infection. Subjects with pressure ulcers had statistically significantly lower fat-free mass component indices compared to those with diabetic foot syndrome and venous ulceration. The subjects with pressure ulcers had significantly lower values of body composition components compared to those with diabetic foot syndrome and venous ulcers. In the group of patients with pressure ulcers, the lowest values of albumin (3.20 g/dL), hemoglobin (10.81 g/dL), and nutritional risk index (NRI) (88.13 pts.) scores were confirmed. Subjects with pressure ulcers with limited self-care presented a non-physiological nutritional status, indicating a risk of malnutrition. Local actions related to wound treatment should be preceded by a general examination, considering the state of augmented nutrition with the use of electrical bioimpedance.

## 1. Introduction

Skin continuity lesions that do not follow the physiological stages of healing in an orderly and timely manner, stopping in the inflammatory phase of healing and thereby not reducing the area of damage over several weeks, are conventionally termed chronic wounds. Chronic wounds occur in 1–2% of the population and are mainly related to blood flow disorders in the vessels of the lower limbs. Leg ulcers are most often diagnosed in patients with chronic venous insufficiency and arterial ischemia. Malnutrition develops more often in the elderly than in the general population. The coexistence of chronic wounds and malnutrition suggests a potential causal relationship [1]. There is an increased risk of chronic wounds with age. It is estimated to be 1.69% for people over 65 and ranges from 0.87% to 3.38% for people over 80. Most chronic wounds are of vascular origin. Approximately 57–80% of leg ulcers are caused by venous insufficiency, 10–25% by atherosclerosis, and 5–2% by diabetic angiopathy [2]. Wound management is multifactorial and costly; it requires interdisciplinary management and intensified medical and nursing care, and is of great economic importance to public health. According to the definition proposed by ESPEN experts (European Society for Clinical Nutrition and Metabolism), malnutrition is a condition resulting from the lack of absorption or lack of consumption of nutrients, predisposing to changes in body composition (decrease in fat-free mass—FFM) and body cell mass (BCM), and thus leading to impairment of the physical and mental functions of the body, at the same time adversely affecting the outcome of treatment of the underlying disease [2,3,4].

Several researchers have shown that weight loss and poor nutrition were associated with a higher risk of pressure ulcers [5,6]. The research of Szewczyk et al. [7] showed that people with venous leg ulcers are much more likely to suffer from malnutrition compared to age-matched controls. In addition, two other studies conducted on people in long-term care facilities indicated that a 5% weight loss over 30 days was associated with a higher risk of death [6,8].

People aged 65 and over are the fastest-growing population. It is expected that in 2050, the improvement in survival will extend the average lifespan by about 5 years for the world population, which in 2019 was 72.6 years [9]. With aging, the risk of malnutrition increases disproportionately due to physical, psychological, social, or economic limitations. People with chronic wounds often show signs of malnutrition not only due to the symptoms of the underlying disease itself but also due to other sociodemographic conditions. Assessment of nutritional status and treatment of possible nutritional deficiencies should be standard in home care. Available data underline the importance and necessity of using the recommended screening methods. It has been shown that the measurement of body weight or waist-to-hip ratio alone is not sufficient to assess the nutritional status of patients with wounds [1,10,11].

The condition for a reliable and accurate nutritional diagnosis is its connection with the parameters of the nutritional status assessment at the basic level (anthropometric measurements, clinical assessment) and in-depth (biochemical tests, bioelectrical impedance) [12,13].

After analyzing the PubMed, Scopus, and Termedia databases, no original or review papers on the assessment of the nutritional status of patients with chronic wounds using bioelectrical impedance and standard methods were found. The current research niche regarding the combination of these methods in relation to the monitoring and co-treatment of hard-to-heal wounds has prompted the authors to focus on this issue. The aim of this study was to analyze the current nutritional status of people with chronic wounds.

## 2. Materials and Methods

### 2.1. Ethics

The study protocol was approved by the ethics committees of the involved institution (Bioethics Commission at the University of Rzeszow: Resolution No. 4/03/2019). In addition, the guidelines of the Declaration of Helsinki were followed in the course of the study. Participants were informed about the purpose of the study and could withdraw at any time without giving any reason.

### 2.2. Subjects

**Figure 1:**
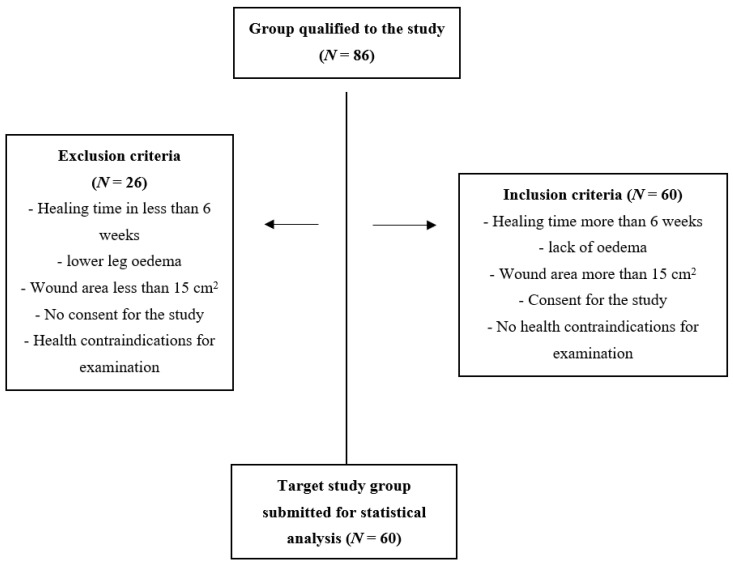
Graphical description of the selection of the study group.

In the designed observational-prospective study, 86 people were qualified, of which 26 patients were excluded due to the occurrence of at least one of the exclusion criteria (treatment duration before 6 weeks, health contraindications for bioelectrical impedance (BIA), withdrawal from the study). Sixty fully completed research questionnaires were submitted for statistical analysis based on the adopted selection criteria (minimum incomplete tissue damage, lack of oedema, wound over 15 cm², wound treated for more than 6 weeks (except for patients with diabetic foot ulcers (DFU), wound duration over 14 days), consent to participate in the study, and mental state allowing for an interview for the purposes of the study; patients were divided into 3 groups of 20 people each: I—people with pressure ulcers (PI—pressure injury), II—those with wounds in the course of DFU, and III—those with venous wounds (VLU—venous leg ulcer) (Figure 1). The tests were carried out at the Wound Treatment Clinic of Fr. B. Markiewicz Podkarpackie Oncology Center in Brzozów (Poland) from February 2021 to September 2022.

### 2.3. Assessments

Research conducted using bioelectrical impedance and standard methods in professional nursing care at home may result in positive economic effects. Multidirectional actions in the form of lowering the costs of treatment and hospitalization, decreasing social isolation of patients, improving the quality of life and care, or unifying standards can contribute to potential profits. In addition, opportunities are created for the nursing staff to increase professional prestige in society while emphasizing the fact that omission or incorrect diagnosis of the nutritional status leads to dangerous issues, unquestionably complicating the treatment process, and in extreme cases, leading to the patient’s death from cachexia rather than from a coexisting wound.

### 2.4. Statistical Analysis

Statistical analysis was performed with Statistica 13.3 by StatSoft. The one-way analysis of the variance ANOVA test was used to compare the results in three groups, defined on a quantitative scale, and Tukey’s test was used as the post-hoc test. Pearson’s chi-square test was used to compare the results in three groups, defined on a qualitative scale. The multiple regression analysis test was used to analyze the influence of several factors simultaneously on the value of a given parameter. The level of statistical significance was *p* < 0.05.

### 2.5. Data Collection

The data were collected using a scientific research protocol developed for the purpose of the study, consisting of four parts (questionnaires). The first part was a questionnaire concerning sociodemographic data, wound assessment, and patient’s capacity in the Barthel scale [14]. Wounds were assessed depending on the etiology (Wagner classification [15]: I°—superficial ulceration, II°—ulceration with inflammation of the skin and subcutaneous tissues, III°—as above and, additionally, inflammation of the bones and phlegmon of the foot, IV°—limited dry or moist necrosis, and V°—extensive necrosis, indications for amputation), depth of structural damage (National Pressure Injury Advisory Panel—NPIAP [16]: I°—epidermal damage, II°—partial-thickness damage, III°—full-thickness damage, and IV°—deep tissue damage, including tendons and bones), and appearance (red yellow black—RYB classification [17], red—granulation tissue, yellow—fibrin and liquified necrosis, black—dry necrosis). The second part included collecting the results of biochemical tests of venous blood (morphology, albumin, and CRP—C-reactive protein). The third part was the assessment of nutritional status using the MNA scale (Mini Nutrition Assessment) [18]. The last part was a questionnaire in which the results of the (BIA) were recorded: body weight, height, and body mass index (BMI), as well as selected components of body composition: body-fat mass, fat-free mass, total body water, muscle mass, phase angle, and basal metabolic rate. The nutritional indicators were calculated as follows: (BMI) was calculated as weight (kg)/height (m^2^) (kg/m^2^); body cell mass index (BCMI), BCM (kg)/height (m^2^) (kg/m^2^); skeletal muscle index (SMI), SM (kg)/height (m^2^); fat mass index (FMI), FM (kg)/height (m^2^) (kg/m^2^); and fat-free mass index (FFMI), FFM (kg)/height (m^2^) (kg/m^2^). The phase angle (PA) was calculated using the following formula: PA = tangent arc (Xc/R) × 180/π [19].

Nutritional status was assessed based on such elements as albumin, Nutritional Risk Index (NRI) [20], which is the resultant of albumin concentration and current body weight; results obtained in the MNA scale; and PA obtained from the bioimpedance measurement. In addition, all results were summarized in a summary table and their correlation was compared (PA vs. MNA/NRI/Albumin).

Assessment of the condition of the subjects related to self-care capacity and verification of wounds was carried out by a designated member of the team with medical education, authorized to assess health and physical examinations and experienced in performing BIA. Blood biochemical tests in all patients were performed in one laboratory (albumin: 3.5–5.2 g/dL; (HGB) hemoglobin: men—13–18 g/dL, women—12–16 g/dL; and CRP 0–5 mg/L). Body composition was measured using the BIA-101 impedance analyzer (Akern SRL, Pontassieve, Florence, Italy). The measurement was made with the tetrapolar (8-electrode) system in the contralateral system (measuring current amplitude 800 uA, sinusoidal, 50 kHz), in the morning (7:00–12:00), in the supine position, abducting the upper (30°) and lower (45°) limbs, on an empty stomach, after a 5-min rest, with the place of electrode attachment washed with alcohol. The location of the wounds was not an obstacle to standard electrode placement. The equations used by the software to assess the specific parameters are restricted property of the company. Body weight and height were measured using a RADWAG C315 electronic personal scale with a height gauge. All subjects with DFU and VLU were subjected to standard measurements, while in subjects with a significant self-care deficit, the Broca index was used to calculate body weight (standard weight (kg)= height (cm)—100). To ensure high reliability of the obtained results, two measurement cycles were performed with disposable electrodes (Biatrodes, Pontassieve, Fl, Italy: single electrode impedance—25–30 Ω, compliance with Directive 93/42/ECC and ISO 10993-1:2003), which were placed on the dorsal surface of the upper (wrist) and lower (ankle) limbs. All measurements were performed according to guidelines described by other authors [21,22,23,24,25]. BIA analysis included fat mass (FM) (kg and %), fat-free mass (FFM) (kg and %), muscle mass (MM) (kg and %), total body water (TBW) (L and %), intra- and extracellular water (ICW and ECW) (%), body cell mass (BCM) (kg), skeletal muscle mass (SMM) (kg), appendicular skeletal muscle mass (ASSM) (kg), and standardized phase angle (SPA) (◦). All obtained results were applied to the authors’ scientific and research protocol. In order to detect significant differences between the subjects, the obtained results were subjected to statistical analysis as the last stage of the study.

### 2.6. Characteristics of the Respondents

Statistical analysis was carried out in a group of 60 people divided into 3 groups according to the type of wound. There were 20 people in each group. Group I consisted of patients with pressure ulcers (PI), group II with DFU, and group III with VLU. The mean age of the patients was 70.38 years. The youngest person was 32 years old and the oldest 98 years old. The standard deviation was 13.34 years.

In group I, there were 10 women and 10 men. There were more men in group II (75.0%) and more women in group III (80.0%). Differences in the number of women and men in the three groups were statistically significant (*p* = 0.002).

Each of the patients had from one to a maximum of four chronic diseases. The most common diseases were hypertension (28.2–41%), diabetes (20–29%), and heart failure (6.9–10%). In each of the three groups, there were more people living in villages than in cities. Most of the respondents were married and widowed. The respondents most often lived with their families, children, or spouses. There were 10.0%, 20.0%, and 25.0% of people living alone in successive groups. More than half of the respondents in each group had primary or vocational education, with 20.0%, 10.0%, and 5.0% having higher education in the following groups (Table 1).

## 3. Results

### 3.1. Wound Characteristics

Taking into account the epidemiology of wounds, the assessment was made using clinical tools used in practice (color classification, NPIAP classification, and Wagner classification). The subjects from the three groups (I–III) did not differ in terms of the results obtained in the RYB classification, although this difference was close to the threshold of significance (*p* < 0.001). However, the location of the wound differed in the three groups. Depending on the etiology, the wounds were typically located in the VLU, in the lower leg area, while in the DFU group, the wounds were located in the area of the toes, foot, and heel. In group I, the location of pressure ulcers was more diverse, although they were most often located in the sacrum, iliac spines, and heels (Table 2).

Only the level of wound exudate, assessed on a scale of 0–4 points, was not significantly different in the three groups. The results of the Barthel scale were the lowest for people from group I (24.50 points); they were significantly lower compared to those from groups II (71.75 points) and III (77 points), similarly to the results of the MNA scale (I—14.73 points, II—20.48 points, III—21.85 points). The results of the NPIAP scale were the highest in group I (deep tissue damage) and significantly higher compared to the results obtained by the subjects from groups II and III. Pain intensity (5.45 points) was the highest, and the period of wound occurrence (10.23 years) was the longest among people from group III. These results were significantly higher compared to group I (2.95 points/0.87 years) and II (3.15 points/1.21 years). The wound area was the smallest in group II (21.60 cm^2^), significantly smaller than in groups I (89.25 cm^2^) and III (94.60 cm^2^) (Table 3).

### 3.2. Lab Tests

In the course of the study, the nutritional status was assessed on the basis of direct blood biochemical components, such as albumin concentration, while calculating the NRI index, and indirect components, such as hemoglobin and CRP levels.

During the examination, selected biochemical components of venous peripheral blood were assessed, such as CRP, hemoglobin, and the concentration of albumin required to calculate the NRI index. Selected biochemical components are sensitive markers of inflammation and malnutrition.

The subjects from group I, compared to those from groups II and III, had the lowest albumin values (I—3.20 g/dL, II—3.79 g/dL, and III—3.90 g/dL) and the lowest NRI scores (I—88.13 pts., II—98.58 pts., and III—100.26 pts.). Subjects from group I also had statistically significantly (*p* < 0.001) lower hemoglobin values (10.81 g/dL vs. 12.19 g/dL) (Table 4).

### 3.3. Wound Condition, Lab Measurement, and Test Results

Individual parameters of biochemical tests changed significantly depending on the condition of the wound and its characteristics. The following positive relationships were found between the wound condition and the results of laboratory tests: The longer the wound exists, the higher the scores on the Barthel scale; the higher the intensity of pain, the higher the scores on the MNA scale. The greater the level of effusion, the more the subjects reported more pain and obtained higher CRP values. The more points the subjects scored on the NPIAP scale, the higher their CRP value. The following negative associations between the condition of the wound and the results of laboratory tests have been shown. The larger the wound surface, the lower the albumin and hemoglobin values obtained by the subjects. The higher the level of exudation, the lower the albumin and hemoglobin values, and the lower the NRI scores. The higher the NPIAP score, the lower the Barthel score and the MNA score, the lower the level of albumin and hemoglobin, and the lower the score on the NRI scale (Table 5).

### 3.4. Electrical Bioimpedance Measurements

Compared to patients from groups II and III, patients from group I with pressure ulcers had statistically significantly lower body weight, FFM, BCM, PA, ICW%, MM, MM %, Mbasale, BMI, BCMI, SMI, SMM, and FFMI indices, and significantly higher Rz and ECW% indices. Patients from group II, compared to patients from group III, had higher indices for body height and FFM %, and lower indices for age and FM %. Patients from group III had lower results for the TBW % parameter and higher results for the FMI parameter compared to patients from groups II and I. Patients from group I had statistically significantly lower results compared to patients from group II for TBW and ASMM parameters. Patients from group III had statistically significantly higher scores compared to patients from group I for FM and SPA parameters (Table 6).

Analyzing all the subjects together, a significant, strong correlation was found between PA and MNA, NRI scales, and the results of the albumin measurement (*p* < 0.001, R > 0.5) (Table 7).

### 3.5. Multiple Regression Model in the Group of Subjects with Pressure Ulcers

Observations related to the increased risk of malnutrition in the group of patients with pressure ulcers prompted a detailed analysis of selected variables that could determine changes in body composition parameters assessed by BIA. The following variables were selected from the analyzed variables: peripheral blood biochemical values (albumin, CRP, and hemoglobin), self-care level (according to the Barthel index), pain (according to NRS), and level of indices (MNA and NRI). The above data were analyzed with the components of body weight (FFM, FM, BCM, PA, TBW, MM, and SMI) in the group of patients with pressure ulcers using a multiple regression model. The table below presents statistically significant data (*p* < 0.05).

First, a regression model was presented (Table 8), containing seven predictors with fat-free mass (FFM) (R2 = 0.70, corrected R2 = 0.52). The entire regression model described was statistically significant (F = 4.04 *p* = 0.017). A detailed analysis of partial correlations showed that the influence of pain on the FFM value was the strongest among the analyzed factors (*p* = 0.015). The impact of other factors turned out to be statistically insignificant. Pain intensity was positively correlated with the FFM score (R = 0.63).

The regression model (Table 9), containing seven predictors, explained 74.0% of the variability in muscle mass (MM) (R2 = 0.74, corrected R2 = 0.59). The entire regression model described was statistically significant (F = 4.99 *p* = 0.007). A detailed analysis of partial correlations showed that the strongest of the analyzed factors was the effect on pain MM (*p* = 0.020) and MNA (*p* = 0.049). These factors correlated positively with MM. The impact of other factors turned out to be statistically insignificant.

The regression model (Table 10), containing seven predictors, explained 69.0% of the variance with the skeletal muscle index (SMI). The entire regression model described was statistically significant (F = 3.78 *p* = 0.021). A detailed analysis of partial correlations showed that the influence of pain on the SMI value was the strongest among the analyzed factors (*p* = 0.032). The impact of other factors turned out to be statistically insignificant. Pain intensity positively correlated with the SMI score (R = 0.57).

When verifying the remaining variables using the seven-variable regression model, the selected factors did not affect the value of BCM (F = 2.31, *p* = 0.096), TBW (F = 1.05, *p* = 0.0445), and PA (F = 1.37, *p* = 0.0298). In the assessment of fat mass (FM), the regression model was not statistically significant. The impact of pain on the FM value was demonstrated (R = 0.57, *p* = 0.034), but this relationship did not determine the significance of the entire model.

## 4. Discussion

The problem of malnutrition is complex and multifactorial, combining physiological, psychosocial, and economic determinants. Identification and assessment of the variables responsible for their formation is therefore scientifically and clinically justified [12]. There is much scientific evidence that targeted nutritional support of people at risk reduces the incidence of the above-mentioned diseases. The results therefore justify the importance and role of screening among patients suffering from various diseases, including hard-to-heal wounds [16,26,27,28].

The subject of the research is extremely inspiring, as it contributes to the growing popularization of nutrition and draws attention to the roles and wide range of possibilities facing medical personnel who want to combine theoretical knowledge with practical clinical management. In practice, the frequency of nutritional disorders is underestimated, too often minimized, and the assessment is often omitted in the routine examination of patients (the answer to the above problem seems to be multifactorial and multithreaded, which can also be a separate discussion) [29]. For years, expert groups and scientific societies have been recommending the assessment of the nutritional status and the risk of malnutrition as basic and routine elements of examining and assessing the patient’s condition [16,28,29,30]. Our study focused on the assessment of enhanced nutritional status in three groups of patients with chronic wounds. It was assumed that people with limited self-care and deep damage to the skin and subcutaneous tissues, compared to other patients, will be at higher risk of malnutrition.

In their study, Crogan and Pasvogel [31], examining 311 residents of three nursing homes, noted that as many as 38.6% of the respondents were malnourished. This had a negative impact on both their functional status (the ability to maintain hygiene, eat independently, or use the toilet) and psychosocial well-being. In our study, it was confirmed that the lower degree of independence of the respondents and their need for care on the Barthel scale predisposed to malnutrition; the lowest results were recorded in the group of people with pressure injury, compared to those from the groups with wounds in the limbs. The relationship between the risk of malnutrition, determined based on the MNA scale, and tissue damage according to the NPIAP was confirmed. The subjects from group I (PI), whose wounds were classified at a higher degree in the NPIAP scale, showed a higher risk of malnutrition in the MNA scale, noting the number of points obtained. The results cited are reflected in studies by other authors. Lamgham-Henken et al. [32] used the MNA tool on patients with pressure injury and found that only 3 of the 23 subjects were considered well-nourished, while 20 were considered at risk of malnutrition, and Hengstermann et al. found that the nutritional status of patients with pressure ulcers was significantly reduced compared to patients without pressure injury [33,34]. On the other hand, in order not to be limited only to pressure injuries, an interesting study was conducted by Szewczyk et al., who showed a significantly higher malnutrition rate of 68.0% in 37 patients with leg ulcers, with a rate of 35.0% also in the group comparative study of vascular patients without wounds, which means that further studies, extended to include a larger group, are necessary [2,7]. The research by Allen et al. confirmed the validity of the thesis that the greater the degree of injury in the NPIAP scale, the greater the risk of malnutrition [35], which showed that individualized assessment and planning of nutrition in the elderly with pressure sores according to NPIAP II or III degree, taking into account other energy inputs, were associated with improved wound healing compared to standard nutritional plans (37% vs. 23.4%, *p* < 0.05) [28]. The percentage of patients suffering from pain was very high, with chronic wounds having a negative impact on quality of life and wound healing [36]. Pain promoted the development of malnutrition, especially through its negative effect on appetite. The relationship between chronic, non-cancerous pain and loss of appetite was reported by Bosley et al. [37], while the hypothetical relationship between malnutrition and chronic pain in wound patients is still poorly understood. In our study, the pain component was also examined, showing that the intensity of pain and the duration of the wound was the greatest/longest among patients from group III (VLU) compared to patients from groups I and II, which gives grounds to confirm the validity of the hypothesis regarding the relationship between chronic pain and the coexisting problem of malnutrition among patients with a chronic wound. It is also worth noting the observed time of occurrence of wounds in group III, which is undoubtedly associated with the generation of high costs of treatment as well as the risk of complications and pathological changes within the wound [38,39,40]. The area of wounds was the smallest in group II, significantly smaller than in groups I and III, which is beyond doubt in relation to the different etiologies of the wounds in the study groups and their exact location related to this. Although the resulting changes may seem small and harmless visually, extreme caution and clinical vigilance should be exercised due to the risk of damage to bone structures that are typical in the course of DFU. In a small amount of subcutaneous tissue, the proximity of important anatomical structures (tendons, bones) may predispose to severe complications, including sepsis and death; therefore, they should not be marginalized [41].

In our study, patients from group I (PI) compared to patients from group II (DFU) and group III (VLU) obtained the lowest albumin concentration and the lowest NRI scores. Patients from group I, compared to patients from group II, also had statistically significantly lower hemoglobin values. The authors of other studies suggest that the level of serum albumin is questionable and not useful as a marker of possible malnutrition due to variables such as age or general condition of the patient; therefore, diagnosis and further management should not be excessively dependent on its level, while stressing the need further research in this direction [42,43]. Interesting and completely different observations are described by Frangos et al. [44] and Eckart et al. [45]. The first of these noted that albumin concentrations are strongly correlated with anemia in the elderly, based on a study group of over 390 patients, where hematological parameters such as hemoglobin, albumin, CRP were assessed, proving that the incidence of anemia (defined as HGB <12 g/L) was 39.3%. Patients with anemia were more often malnourished or at risk of malnutrition according to MNA-SF (*p* = 0.047), with lower serum albumin concentration (*p* < 0.001), which is consistent with our observations [44]. The second author, in his prospective study, assessed the relationship between nutritional status and inflammation with low serum albumin levels and 30-day mortality in a cohort of over 2000 patients. The analysis confirmed a strong relationship between three parameters independently predicting mortality. Elevated parameters of inflammation and high nutritional risk were independently associated with hypoalbuminemia, and combining them during the initial assessment of patients facilitates the potential risk of future death. Although the studies concerned emergency department patients, they are a great example of the nutritional problem, which also undoubtedly occurs among patients with wounds, and may be a prelude to further scientific research in this direction [45]. Furthermore, an interesting correlation was found: The longer the duration of the wound presence, the higher the scores on the Barthel scale, the higher the severity of pain, and the higher the scores on the MNA scale. Hypothetically, the obtained correlation can be explained on several levels, where the first of them may be the fact that the moment of wound formation generates huge energy losses (micro and macro components), usually associated with the random health situation of the patient. The patient’s self-care capacity drops dramatically when dealing with a new situation/adverse event, while over time the system strives for a kind of balance and homeostasis, and the patient, thanks to the support of key people and medical staff in accordance with the PCC (Person-Centered Care) concept, learns functions with the resulting disease entity, which is a chronic wound, striving to heal it. This may explain the fact that higher scores on the Barthel/MNA scale are obtained along with a longer duration of non-healing wounda [46,47].

Attention should be given to the exudate component; it is important in wound healing and/or management because the exudate is not only water but also other components such as platelets, plasma proteins, glucose, growth factors, and waste products, which can contribute and cause higher CRP values, infection, pain, hypoalbuminemia, skin maceration, and dehydration, including malnutrition, which is consistent with our own observations [48].

A review of the available resources of the current literature failed to find similar studies evaluating individual parameters of electrical bioimpedance in patients with a chronic wound. Noteworthy, however, are the studies of Pigłowska et al., who conducted an analysis of the functional state and nutrition between the two surveyed groups of seniors in a social environment and those staying in long-term care facilities (*N* = 200). The assessment was performed using a comprehensive geriatric assessment, anthropometric measurements, the MNA questionnaire, and BIA analysis. The obtained results indicated significant discrepancies in the functional and nutritional status between the two study groups, indicating, at the same time, that the key element in ensuring functional independence of seniors is maintaining proper nutritional parameters [49].

Referring to the results of our study, it was shown that patients from group I with pressure ulcers had statistically significantly lower FFM, BCM, PA, ICW%, MM, MM%, Mbasale, BMI, BCMI, SMI, SMM, and FFMI indices, in comparison with the subjects from groups II (DFU) and III (VLU), which correlates with the previously discussed results and is in line with the research conclusions of Pigłowska et al. [49].

The subjects from group III (VLU) presented higher results compared to subjects from group I (PI) and group II (DFU) for the FM and FMI parameters. The discussed parameters are related to adipose tissue, namely, its total mass. Changes in body composition that progress with age may predispose not only to cachexia but also to obesity (along with its negative health consequences), including sarcopenic obesity.

This is diagnosed in patients who meet at least one criterion for obesity: BMI > 35 kg/m^2^, body fat (FM%) > 35%, or fat mass index (FMI) calculated using the formula FM (kg)/body height (m)^2^ > 9.5 kg/m^2^ [50,51]. Interestingly, the above criteria are practically fully met by group III (VLU) (BMI: 34.49, FM: 36.28, FMI: 13.27), patients with a venous ulcer type wound, at the same time refuting the established opinion and the prevailing myth that malnutrition cannot coexist with obesity; therefore, fortification of an additional diet in such patients is not necessary and can be abandoned [52].

A strong correlation was observed between PA and MNA, NRI and albumin scores (*p* < 0.001, R > 0.5). This means that people with a higher phase angle (PA) also had a better nutritional status. People with a lower phase angle had a higher risk of malnutrition (MNA), as well as the results obtained on the NRI scale or the level of albumin concentrations.

Application of BIA and PA in the assessment of nutrition in the population of healthy adults and children was used by Więch in his research. Despite the fact that the study covered healthy people, an interesting observation was that in the oldest age group, the value of potential malnutrition oscillated at 11%, which confirms the concept that in people of geriatric age, the stability of cell membranes may be lower, and therefore there is a relatively higher risk of malnutrition [53]. Research by English-language authors also confirmed the fact that the PA measured in a non-invasive way using bioelectrical impedance analysis may be potentially new, objective, and useful as a clinical practice indicator of proper nutritional status, positively correlating with other markers of this condition [54,55,56]. In addition, it can be used prognostically in severe disease debilitating organisms, as well as being a helpful tool for identifying malnourished patients from groups at high risk of death. Buscemi et al. [57] performed an observational-prospective study on a group of 225 patients aged ≥60 years admitted to three selected departments of the University Hospital of Palermo with a follow-up period of 48 months. Anthropometric, blood biochemical (including albumin), and (BIA) measurements were performed and assessed with the MNA questionnaire. The results obtained indicated that 40% of the participants (*n* = 90) died at the end of the follow-up. Significant correlations were found between PA, MNA score, age, and gender regarding mortality. Patients with the lowest PA score <4.6° had higher mortality; similarly, survival curves showed that low MNA scores <22 points correlated with a higher death rate.

Undoubtedly, after the age of 45, the skeletal muscle mass gradually decreases, which in turn may lead to impairment of motor functions, deterioration of the quality of life, or disability. An important parameter assessed as a reference point was the SMI index, according to the Third National Health and Nutrition Examination Survey (NHANES III) [58]. Based on the guidelines of the European Working Group on Sarcopenia in Older People (EWGSOP), BIA is considered a recommended tool for estimating skeletal muscle mass [58,59].

Our study indicated differences in muscle mass parameters (SM, SMI, ASMM) in individual groups. The values obtained in group I (PI) were the lowest (SM—23.42 kg, SMI—8.09 kg/m^2^, ASMM—18.41 kg) compared to groups II and III. In a study by Jenssen et al. [58] and Cruz-Jentoft et al. [60], the authors defined the usual cut-off point used to define sarcopenia with SMI: moderate sarcopenia when the SMI was between 8.51 and 10.75 kg/m^2^ (men) or 5.76 and 6.75 kg/m^2^ (women), and severe sarcopenia when SMI was ≤8.50 kg/m^2^ (men) or ≤5.75 kg/m^2^ (women). Referring to the results of our study, where group I (PI) consisted of both women and men, women, based on the average SMI score, oscillated within normal limits, while men with an SMI score of 8.09 kg/m^2^ obtained a result indicating the likelihood of severe sarcopenia. The regression analysis showed that the influence of the selected variables on the value of FFM, MM, and SMI was positive, clearly not differing in relation to others, except for the predictor of pain, which was statistically visible in all tables concerning the discussed values.

Transferring the above data to clinical practice, and based on own observations, the method of electrical bioimpedance unquestionably fits into the scheme of screening assessment of body composition as well as estimating the risk of sarcopenia in elderly patients with concomitant chronic wounds, while being a useful tool in the hands of clinicians with specialist–holistic care.

### Limitations

The presented study has several limitations. Firstly, it was conducted in one center within one province. The study included a small number of patients. Extending the research to multiple centers, unifying the study group in terms of individual types of wounds, as well as correlation of comorbidities in future studies, or post-treatment follow-up may be an interesting option to include, which could additionally bring wider and more valuable results. Nevertheless, this study provides valuable information on nutritional status and is a kind of starting point for further advanced scientific inquiries in this research group.

## 5. Conclusions

Patients with a chronic wound of the etiology of pressure ulcers with limited self-care present a nutritional status deviating from the norm, indicating the risk of malnutrition. Local actions related to wound treatment should be preceded by a general examination, taking into account the state of augmented nutrition with the use of electrical bioimpedance. The BIA method allows one to provide detailed and reliable data on the nutritional status, its individual components, which are useful in the assessment and monitoring of nutritional status. The simplicity of implementation gives ample opportunities for the assessment of patients who are incapable of or limited in self-care, where abandonment or incorrect diagnosis of the nutritional status of patients treated in non-hospital environments may lead to longer treatment times and higher costs. In extreme cases, this may lead to devastation and serious dysfunction of vital systems.

## Figures and Tables

**Table 1:** Characteristics of the subjects.

Parameters		PI—I *N* = 20		DFU—II *N* = 20		VLU—III *N* = 20		Chi-Square Pearson/*p*-Value	
		*N*	%	*N*	%	*N*	%	χ²	*p*
Sex	Female	10	50.0	5	25.0	16	80.0	12.15	**0.002**
	Male	10	50.0	15	75.0	4	20.0		
Place of residence	Rural area	14	70.0	17	85.0	16	80.0	1.37	0.503
	Urban area	6	30.0	3	15.0	4	20.0		
Marital status	Married	9	45.0	14	70.0	8	40.0	7.95	0.242
	Single	3	15.0	3	15.0	2	10.0		
	Widowed	7	35.0	3	15.0	10	50.0		
	Divorced	1	5.0	0	0.0	0	0.0		
People living with the subject	Children	4	20.0	0	0.0	1	5.0	7.26	0.297
	Family	10	50.0	10	50.0	11	55.0		
	Spouse	4	20.0	5	25.0	3	15.0		
	Lonely	2	10.0	5	25.0	5	25.0		
Education level	Primary	8	40.0	7	35.0	8	40.0	4.09	0.665
	Vocational	3	15.0	5	25.0	7	35.0		
	Secondary	5	25.0	6	30.0	4	20.0		
	Higher	4	20.0	2	10.0	1	5.0		
Economic status	National average	2	10.0	5	25.0	1	5.0	5.72	0.221
	Below national average	17	85.0	15	75.0	19	95.0		
	Over national average	1	5.0	0	0.0	0	0.0		
ICD-10	L89	20	100.0	0	0.0	0	0.0	120.0	**<0.001**
	E10.5	0	0.0	20	100.0	0	0.0		
	I83.0	0	0.0	0	0.0	20	100.0		

Abbreviations: PI—Pressure Injury; DFU—Diabetic Foot Ulcer; VLU—Venous Leg Ulcer; International Classification of Diseases (ICD-10). Bold characters indicate significant values (*p* < 0.05).

**Table 2:** Wound characteristics.

Parameters		PI –I *N* = 20		DFU—II *N* = 20		VLU—III *N* = 20		Chi-Square Pearson/ *p*-Value	
		*N*	%	*N*	%	*N*	%	χ²	*p*
RYB classification	Yellow-black	4	20.0	0	0.0	0	0.0	15.24	0.055
	Yellow	2	10.0	4	20.0	3	15.0		
	Red	5	25.0	9	45.0	8	40.0		
	Black	0	0.0	2	10.0	0	0.0		
	Red-yellow	9	45.0	5	25.0	9	45.0		
Wound location	Sacrum	15	75.0	0	0.0	0	0.0	120.0	**<0.001**
	Trochanter	1	5.0	0	0.0	0	0.0		
	Sacrum + heel	4	20.0	0	0.0	0	0.0		
	Toe	0	0.0	6	30.0	0	0.0		
	Heel	0	0.0	4	20.0	0	0.0		
	Foot	0	0.0	8	40.0	0	0.0		
	Medial ankle	0	0.0	0	0.0	1	5.0		
	Lower leg	0	0.0	0	0.0	19	95.0		
	Foot + toes	0	0.0	2	10.0	0	0.0		

Abbreviations: PI—Pressure Injury; DFU—Diabetic Foot Ulcer; VLU—Venous Leg Ulcer; Red Yellow Black classification (RYB). Bold characters indicate significant values (*p* < 0.05).

**Table 3:** Test and scale results.

Parameters	PI—I *N* = 20		DFU—II *N* = 20		VLU—III *N* = 20		One-Way ANOVA F/*p*-Value		Post-hoc (Tukey’s) *p*-Value		
	Mean	SD	Mean	SD	Mean	SD			I-II	I-III	II-III
Barthel (pts)	24.50	26.10	71.75	27.64	77.00	18.88	27.85	**<0.001**	**<0.001**	**<0.001**	0.777
Pain (pts)	2.95	2.68	3.15	2.16	5.45	2.01	7.27	**0.002**	0.959	**0.003**	**0.007**
Time since wound onset (years)	0.87	0.93	1.21	1.75	10.23	10.54	14.69	**<0.001**	0.983	**<0.001**	**<0.001**
Wound area (cm^2^)	89.25	93.87	21.60	16.37	94.60	82.71	6.24	**0.004**	**0.013**	0.971	**0.007**
Exudate (0–4)	2.25	1.02	1.80	1.06	2.20	0.70	1.38	0.259	-	-	-
NPIAP (1–4)	3.18	0.44	2.60	0.62	2.38	0.43	13.49	**<0.001**	**0.002**	**<0.001**	0.339
Wagner scale *			* 1.85	0.88							
MNA (pts)	14.73	4.30	20.48	3.80	21.85	2.22	22.60	**<0.001**	**<0.001**	**<0.001**	0.445

* for DFU; Abbreviations: PI—Pressure Injury; DFU—Diabetic Foot Ulcer; VLU—Venous Leg Ulcer; NPIAP—National Pressure Injury Advisory Panel; MNA—Mini Nutrition Assessment; pts – points. Bold characters indicate significant values (*p* < 0.05).

**Table 4:** Lab/index tests.

Parameters	PI—I *N* = 20		DFU—II *N* = 20		VLU—III *N* = 20		One-Way ANOVA F/*p*-Value		Post-hoc (Tukey’s) *p*-Value		
	Mean	SD	Mean	SD	Mean	SD			I-II	I-III	II-III
Albumin (g/dL)	3.20	0.56	3.79	0.47	3.90	0.38	12.74	**<0.001**	**0.001**	**<0.001**	0.765
Hemoglobin (g/dL)	10.81	1.57	12.19	1.76	11.97	1.55	4.14	0.021	**0.026**	0.072	0.905
CRP (mg/L)	49.40	61.09	33.96	35.96	18.26	21.03	2.66	0.079	-	-	-
NRI (pts)	88.13	8.71	98.58	7.25	100.26	5.65	16.18	**<0.001**	**<0.001**	**<0.001**	0.747

Abbreviations: PI—Pressure Injury; DFU—Diabetic Foot Ulcer; VLU—Venous Leg Ulcer; CRP—C-reactive protein; NRI—Nutritional risk index; pts – points. Bold characters indicate significant values (*p* < 0.05).

**Table 5:** Analysis of the relationship between the condition of the wound and the results of lab measurements and tests.

Variable	Time Since Wound Onset (years)	Wound Area (cm^2^)	Exudate (0–4)	NPIAP (1–4)
Barthel (pts)	r = 0.31	r = −0.24	r = −0.14	r = −0.62
	***p* = 0.018**	*p* = 0.069	*p* = 0.296	***p* < 0.001**
Pain (pts)	r = 0.39	r = 0.12	r = 0.31	r = 0.02
	***p* = 0.002**	*p* = 0.343	***p* = 0.016**	*p* = 0.902
MNA (pts)	r = 0.31	r = −0.12	r = −0.17	r = −0.59
	***p* = 0.017**	*p* = 0.355	*p* = 0.191	***p* < 0.001**
Albumin (g/dL)	r = 0.15	r = −0.26	r = −0.33	r = −0.65
	*p* = 0.243	***p* = 0.044**	***p* = 0.009**	*p* < 0.001
Hemoglobin (g/dL)	r = 0.08	r = −0.30	r = −0.41	r = −0.44
	*p* = 0.526	***p* = 0.020**	***p* = 0.001**	***p* = 0.001**
CRP (mg/L)	r = −0.09	r = 0.16	r = 0.31	r = 0.27
	*p* = 0.475	*p* = 0.209	***p* = 0.016**	***p* = 0.038**
NRI (pts)	r = 0.18	r = −0.25	r = −0.34	r = −0.66
	*p* = 0.178	*p* = 0.053	***p* = 0.008**	***p* < 0.001**

Abbreviations: MNA—Mini Nutrition Assessment; CRP—C-reactive protein; NRI—Nutritional risk index; r—value of Pearson’s linear correlation test; pts – points; *p*—test probability index. Bold characters indicate significant values (*p* < 0.05).

**Table 6:** Electrical bioimpedance measurements.

Parameters	PI—I *N* = 20		DFU—II *N* = 20		VLU—III *N* = 20		One-Way ANOVA F/*p*-Value		Post-hoc (Tukey’s) *p*-Value		
	Mean	SD	Mean	SD	Mean	SD			I-II	I-III	II-III
Height (cm)	168.55	9.69	173.40	6.89	164.45	8.41	5.67	**0.006**	0.171	0.279	**0.004**
Age (years)	71.90	15.56	62.40	10.35	72.55	12.75	3.79	**0.029**	0.063	0.986	**0.044**
Weight (kg)	72.55	20.06	91.15	12.58	94.40	27.92	6.22	**0.004**	**0.020**	**0.005**	0.878
Rz (ohm)	575.31	142.85	441.33	64.22	444.07	94.46	10.52	**<0.001**	**0.001**	**<0.001**	0.996
Xc (ohm)	37.52	15.34	42.42	7.10	38.81	5.80	1.21	0.305	-	-	-
FFM (kg)	48.65	12.03	64.52	9.64	58.12	14.75	8.40	**0.001**	**0.001**	**0.047**	0.237
TBW (L)	38.31	10.37	48.62	7.02	44.68	12.58	5.15	**0.008**	**0.007**	0.131	0.449
BCM (kg)	19.37	7.91	33.17	7.57	28.67	9.57	14.04	**<0.001**	**<0.001**	**0.003**	0.216
FM (kg)	23.90	11.74	26.64	10.34	36.28	16.79	4.82	**0.012**	0.792	**0.013**	0.064
PA (°)	3.78	1.14	5.55	0.95	5.09	0.73	18.41	**<0.001**	**<0.001**	**<0.001**	**0.295**
FM (%)	31.48	10.98	28.80	8.86	37.19	7.95	4.19	**0.020**	0.638	0.140	**0.017**
FFM (%)	68.52	10.98	71.20	8.86	62.82	7.95	4.19	**0.020**	0.638	0.139	**0.017**
TBW (%)	53.48	7.72	53.56	5.85	48.05	6.15	4.54	**0.015**	0.999	**0.032**	**0.029**
ECW (%)	59.75	7.77	48.36	4.84	50.57	3.99	21.97	**<0.001**	**<0.001**	**<0.001**	0.451
ICW (%)	40.25	7.77	51.65	4.84	49.44	3.99	21.97	**<0.001**	**<0.001**	**<0.001**	0.451
MM (kg)	23.42	8.64	31.46	5.42	31.11	11.54	5.22	**0.008**	**0.016**	**0.022**	0.992
MM (%)	32.50	9.41	34.72	5.60	33.18	8.24	0.42	0.662	-	-	-
Mbas.(kcal)	1311.79	229.43	1712.21	219.65	1581.21	277.50	14.06	**<0.001**	**<0.001**	**0.003**	0.214
BMI (kg/m^2^)	25.41	6.10	30.43	4.73	34.49	7.98	10.08	**<0.001**	**0.042**	**<0.001**	0.121
BCMI (kg/m^2^)	6.78	2.50	10.97	2.13	10.42	2.53	18.08	**<0.001**	**<0.001**	**<0.001**	0.749
SMI (kg/m^2^)	8.09	2.58	10.42	1.35	11.35	3.49	8.19	**0.001**	**0.019**	**0.001**	0.505
SM (kg)	23.42	8.64	31.46	5.42	31.11	11.54	5.22	**0.008**	**0.016**	**0.022**	0.992
ASMM (kg)	18.41	5.72	24.82	3.77	22.92	7.89	5.96	**0.004**	**0.004**	0.055	0.580
FMI (kg/m^2^)	8.41	4.17	9.06	4.07	13.27	5.59	6.39	**0.003**	0.898	**0.005**	**0.016**
FFMI (kg/m^2^)	17.01	3.27	21.40	2.42	21.24	3.52	12.84	**<0.001**	**<0.001**	**<0.001**	0.984
SPA (°)	−1.35	2.38	−0.21	0.85	0.19	0.70	5.53	**0.006**	0.055	**0.006**	0.675

Abbreviations: PI—Pressure Injury; DFU—Diabetic Foot Ulcer; VLU—Venous Leg Ulcer; Rz—resistance; Xc—reactance; FFM—fat-free mass; TBW—total body; BCM—body cell mass; FM—fat mass; PA—phase angle; FM—fat mass; ECW—extracellular water; ICW—intracellular water; MM—muscle mass; BMI—body mass index; BCMI—body cell mass index; SMI—skeletal muscle index; SMM—skeletal muscle mass; ASMM—appendicular skeletal muscle mass; FMI—fat mass index; FFMI—fat-free mass index; SPA—standardized phase angle. Bold characters indicate significant values (*p* < 0.05).

**Table 7:** PA vs. MNA, NRI, albumins—all subjects.

Variables	R	*p*-Value
PA vs. MNA	0.62	**<0.001**
PA vs. NRI	0.59	**<0.001**
PA vs. albumins	0.55	**<0.001**

Abbreviations: PA—phase angle; MNA—Mini Nutrition Assessment; NRI—Nutritional risk index; R—value of Spearman’s rank correlation test; *p*—test probability index. Bold characters indicate significant values (*p* < 0.05).

**Table 8:** Influence of selected variables on the FFM value.

FFM	Multiple Regression						
	R^2^	Corrected R^2^	F	*p* Regression Model	b	Partial Correlation	*p*-Value
Barthel (pts)	0.70	0.52	4.04	0.017	0.26	0.53	0.052
Pain (pts)					2.70	0.63	**0.015**
MNA (pts)					1.40	0.43	0.123
Albumin (g/dL)					−18.29	−0.25	0.390
Hemoglobin (g/dL)					−0.02	−0.00	0.990
CRP (mg/L)					−0.04	−0.22	0.450
NRI (pts)					0.75	0.16	0.589

R^2^—regression model. Corrected R2—regression model, removes extreme values. F—Fisher’s test result. b—regression coefficient. Partial correlation—the Xi variable is correlated with the Y variable after taking into account the influence of all other independent variables (simultaneous influence of all four factors). *p*—significance level. Bold characters indicate significant values (*p* < 0.05).

**Table 9:** Influence of selected variables on the value of MM.

MM	Multiple regression						
	R^2^	Corrected R^2^	F	*p* Regression Model	b	Partial Correlation	*p*-Value
Barthel (pts)	0.74	0.59	4.99	0.007	0.13	0.41	0.140
Pain (pts)					1.69	0.61	**0.020**
MNA (pts)					1.23	0.53	**0.049**
Albumin (g/dL)					5.83	0.12	0.676
Hemoglobin (g/dL)					−1.17	−0.27	0.343
CRP (mg/L)					0.01	0.07	0.820
NRI (pts)					−0.29	−0.09	0.753

Bold characters indicate significant values (*p* < 0.05).

**Table 10:** Influence of selected variables on the SMI value.

SMI	Multiple Regression						
	R^2^	Corrected R^2^	F	*p* Regression Model	b	Partial Correlation	*p*-Value
Barthel (pts)	0.69	0.51	3.78	0.021	0.03	0.27	0.353
Pain (pts)					0.51	0.57	**0.032**
MNA (pts)					0.39	0.52	0.057
Albumin (g/dL)					0.76	0.05	0.868
Hemoglobin (g/dL)					−0.32	−0.23	0.436
CRP (mg/L)					−0.00	−0.13	0.667
NRI (pts)					−0.02	−0.02	0.936

Bold characters indicate significant values (*p* < 0.05).

## Data Availability

The data presented in this study are available on reasonable request from the corresponding author: pwiech@ur.edu.pl.
